# Stem Cell Therapy for Erectile Dysfunction of Cavernous Nerve Injury Rats: A Systematic Review and Meta-Analysis

**DOI:** 10.1371/journal.pone.0121428

**Published:** 2015-04-10

**Authors:** Haitao Shan, Fengzhi Chen, Tao Zhang, Shuhua He, Le Xu, Anyang Wei

**Affiliations:** 1 Department of Urology, Hexian Memorial Affiliated Hospital, Southern Medical University, Guangzhou, China; 2 Department of Urology, Medical Center for Overseas Patients, Nanfang Hospital, Southern Medical University, Guangzhou, China; University of Miami Miller School of Medicine, UNITED STATES

## Abstract

**Introduction:**

Stem cell treatment is a novel therapeutic strategy for erectile dysfunction (ED) patients with bilateral cavernous nerve injury (CNI). The relative animal studies provide important clues to design pre-clinical studies and clinical studies further in the future.

**Purpose:**

This study aims to evaluate the effects and influential factors of stem cell transplantation on ED rats with CNI.

**Materials and Methods:**

We searched PubMed and EBSCO databases published before April 30, 2014 for pre-clinical studies to evaluate the efficacy of stem cell transplantation in the treatment of ED rats with CNI. A systematic review and a planned subgroup analysis were performed to identify whether or not some certain influential factors could bring significant effects on stem cell treatment.

**Results:**

12 studies with 319 rats were enrolled in this meta-analysis. Pooled analysis results confirmed the efficacy of stem cell transplantation. Subgroup analysis results showed that treatment effects were not related to CNI models, follow-up time, stem cell species, stem cell sources, markers and delivery approaches in the transplantation. Uncultured stem cells were poorly effective compared with cultured stem cells. Periprostatic implantation (PPI) with acellular scaffolds could promote cavernous nerve regeneration, but was less effective for smooth muscle cell recovery. Stem cells modified by NGF or BDNF combined with udenafil/bFGF seemed to be more effective than those modified by BDNF alone.

**Conclusion:**

This meta-analysis shows that stem cell therapy can be performed to recover erectile function. Future studies should focus on nerve restoration and vascular cell recovery. The synergistic actions of multiple growth factors following stem cell transplantation should also be considered as beneficial strategies to obtain preferable effects.

## Introduction

Cavernous nerve injured ED is a serious complication of radical prostatectomy. It still occurs although advanced surgical techniques and equipment, such as nerve-sparing robot-assisted procedures, have been applied [1]. Furthermore, it cannot be completely cured with the combined application of vacuum erection device and phosphodiesterase-5 inhibitors (PDE-5i) [2]. Thus, a novel therapeutic strategy should be developed to restore cavernous nerves and rehabilitate erectile function.

Stem cells can undergo self-renewal and differentiate into various phenotypes. Furthermore, they can functionally and structurally regenerate damaged tissues [3]. Hence, stem cell therapies were designed to restore the erectile function of CNI rats, and numerous studies have demonstrated improved function and structure of the penis after stem cell transplantation. Efforts have been devoted to find the underlying mechanism of action and improve the therapeutic effects of stem cells.

Accordingly, a systematic review of the main issues and improvements in this field was performed. The true values of improvements were investigated by pool analysis.

## Systematic Review

At least 24 articles regarding stem cell therapy on rats with CNI had been published before April 30, 2014. 22 articles among these were included by SCI. The impact factors of 14 reports are >3. The characteristics of the published articles and the characteristics of stem cells are shown in Tables 1 and 2.

**Table 1:** Characteristics of the articles published.

Year	First author	Institution	Impact Factor	Model	Stem cell
2004	Bochinski [4]	University of California	3.046	Crush	Allogeneic NESCs
2006	Y Kim [5]	University of Pittsburgh School of Medicine	1.511	Transection	Allogeneic SkMSCs
2009	Fall [6]	Henri Mondor Teaching Hospital France	10.476	Ablation	Allogeneic BMMNCs
2010	Albersen [7]	University of California	3.513	Crush	Autologous ADSCs
2010	Kendirci [8]	Sisli Etfal Training and Research Hospital Turkey	3.696	Crush	Allogeneic BMSCs
2011	Lin [9]	University of California	1.511	Crush	Autologous ADSCs or Allogeneic ADSCs
2011	Lin [10]	University of California	2.424	Resection	Autologous ADSCs
2011	Woo [11]	The Catholic University of Korea	Not SCI	Transection	Allogeneic SkMSCs
2012	Fandel [12]	University of California	10.476	Crush	Autologous ADSCs
2012	SJ Kim [13]	The Catholic University of Korea	0.742	Crush	Allogeneic BMSCs
2012	SJ Kim [14]	The Catholic University of Korea	Not SCI	Crush	Allogeneic BMSCs
2012	Kovanecz [15]	Los Angeles Biomedical Research Institute	3.513	Resection	Heterogeneous SkMSCs
2012	Piao [16]	The Catholic University of Korea	3.513	Crush	Heterogeneous ADSCs
2012	Qiu [17]	University of California	3.513	Radiation	Allogeneic ADSCs
2012	Qiu [18]	University of California	10.476	Crush	Autologous SVF
2013	Jeong [19]	The Catholic University of Korea	2.424	Crush	Heterogeneous ADSCs
2013	You [20]	University of Ulsan College of Medicine, Korea	3.843	Stretch	Heterogeneous ADSCs
2013	You [21]	University of Ulsan College of Medicine, Korea	2.424	Not described	Heterogeneous BMSCs
2013	IG Kim [22]	The Catholic University of Korea	4.254	Crush	Heterogeneous ADSCs
2013	Choi [23]	CHA University, Seoul, Korea	4.67	Crush	Heterogeneous TDSCs
2013	Ying [24]	Zhongnan Hospital,Wuhan University	2.293	Crush	Allogeneic ADSCs
2014	Ying[25]	Zhongnan Hospital,Wuhan University	2.293	Resection	Allogeneic ADSCs
2014	Miyamoto [26]	Hiroshima University, Hiroshima, Japan	3.513	excision	Heterogeneous BMCD133+
2014	Lee [27]	Gangnam Severance Hospital, Seoul, Korea	4.254	Crush	Heterogeneous ADSCs

NESCs:neural embryonic stem cells; SkMSCs:skeletal muscle-derived stem cells; BMMNCs:bone marrow mononucleated cells;ADSCs:adipose tissue-derived stem cells; BMSCs:bone marrow stem Cells; SVF:adipose-derived stromal vascular fraction; TDSCs:testis-derived stem cells; BMCD133^+^:Bone Marrow Derived CD133^+^ Cells.

**Table 2:** Characteristics of stem cells.

Year	First Author	numbers	Label	Modification	Follow-up Time	Fate
2004	Bochinski [4]	5 X10^3^	GFP	BDNF transduction	3 months	undetectable
2006	Y Kim [5]	1X10^6^	lacZ	none	2 weeks / 4 weeks	detected
2009	Fall [6]	1X10^7^	PKH-26	none	3 weeks / 5 weeks	detected
2010	Albersen [7]	1X10^6^	Edu	none	4weeks	very few
2010	Kendirci [8]	0.5 X10^6^	GFP transgene	p75LNGFR selection	4weeks	undetectable
2011	Lin [9]	1X10^6^	Edu	none	7days	time-dependent disappearance
2011	Lin [10]	unclear	Edu	none	3 months	detected
2011	Woo [11]	1X10^6^	PKH-26	none	4 weeks	detected
2012	Fandel [12]	2X10^6^	Edu	none	4 weeks	time-dependent disappearance
2012	SJ Kim [13]	1X10^6^	PKH-26	none	4 weeks	detected
2012	SJ Kim [14]	1X10^6^	unclear	BDNF transduction	4 weeks	detected
2012	Kovanecz [15]	1X10^6^	unclear	Oral sildenafil	42days	undetectable
2012	Piao [16]	1X10^6^	PKH-26	BDNF	4 weeks	quantify
2012	Qiu [17]	1X10^6^	Edu	none	17 weeks	very few
2012	Qiu [18]	2X10^6^	none	none	12 weeks	No mentioned
2013	Jeong [19]	1X10^6^	none	BDNF Oral udenafil	4 weeks	No mentioned
2013	You [20]	1X10^6^	CELL STALKER	None	4 weeks	detected
2013	You [21]	1X10^6^	CELL STALKER	None	4 weeks	detected
2013	IG Kim [22]	1X10^6^	PKH-26	NGF	4 weeks	quantify
2013	Choi [23]	1X10^7^	CM-DiI	none	4 weeks	detected
2013	Ying [24]	1X10^6^	none	none	3 months	No mentioned
2014	Ying [25]	1X10^6^	none	none	3 months	No mentioned
2014	Miyamoto [26]	1X10^6^	none	none	12 weeks	detected
2014	Lee [27]	1X10^6^	PKH-26	BDNF, bFGF	4 weeks	detected

### Fate and mechanism of action of stem cells transplanted

Nine studies were conducted in USA. Among these studies, seven were from the University of California. In one study, neural embryonic stem cells (NESCs) were investigated. In the six other studies, therapeutic effects, mechanism of action and fates of adipose tissue-derived stem cells (ADSCs) were further explored. Moreover, these studies are very coherent.

Bochinski [4] was the first to report and confirm the effects of stem cell transplantation on rats with CNI. In his study higher amounts of nitric oxide synthase (NOS)-containing nerve fibres were found after brain-derived neurotrophic factor (BDNF)-transfected NESCs were transplanted compared with those in the sham group. The following two interesting phenomena were also described: (1) No stem cell marker was found in the tissue specimens three months after transplantation was performed. (2) The stem cells injected into the major pelvic ganglion (MPG) or cavernous body brought similar effects on erectile function recovery.

Albersen [7] reported that ADSC-derived lysate could restore erectile function in rats with CNI almost as effectively as ADSCs, thus, ADSCs functioned by releasing intracellular preformed substances or by actively secreting specific biomolecules. Since only a few labelled stem cells were found four weeks after injection, he concluded that paracrine was possibly the main mechanism of action involved in stem cell activity.

Lin [9] found that intracavernously injected (ICI) ADSCs left from the penis within days to migrate preferentially to the bone marrow and then to the MPG. He further suggested that disease status, such as CNI, negatively influenced the presence of ADSCs in the bone marrow and likely redirected these cells to injury sites, such as the MPG. Fandel [12] also reported that CNI unregulated stromal cell-derived factor-1 (SDF-1) expression in the MPG, thereby attracting ICI ADSCs. He found at the MPG, ADSCs elicited neuroregenerative effects on the cell bodies of injured nerves, resulting in enhanced erectile response.

Qiu [17] reported that tail vein-injected ADSCs significantly restored erectile function, neuronal nitric oxide synthase (nNOS) expression and cavernous smooth muscle content in irradiated rats. 17 weeks after these cells were injected in the vein, ADSCs remained undetectable in the penis, but were scantly visible in the MPG.

Thus far, the fates of transplanted stem cells have been elucidated. However, in view of only a few ADSCs could be detected four weeks after transplantation, the mechanism by which such a few stem cells elicit the same effects as millions of stem cells directly injected in the MPG remains unclear.

Stem cells injected in the MPG or cavernous body elicited similar therapeutic effects [4]. Whether different delivery approaches have a similar mechanism of action should be elucidated. In two studies conducted by one group from the University of Ulsan College of Medicine (Korea), the therapeutic mechanisms of stem cell delivery approaches, particularly periprostatic implantation (PPI) and ICI, were compared and clarified.

You [20] from the above-mentioned group compared the PPI and ICI effects of ADSCs on rats with CNI. The results showed that both methods could be used to improve erectile function. After stem cell therapy was performed, the expression of nNOS increased slightly in the ICI group without statistical relevance; by contrast, the PPI group and the combined treatment (PPI + ICI) group showed a marginally significant increase in the expression of nNOS (P = 0.08). The combined treatment group also showed a remarkable but not statistically significant increase in the smooth muscle content to a greater extent than either of the treatments administered individually. You suggested that PPI and ICI of ADSCs in CNI rat model were similarly effective in promoting penile erection recovery. Nevertheless, these two techniques might address different types of pathophysiology.

You [21] compared the PPI and ICI effects of bone marrow-derived mesenchymal stem cells (BMSCs) on CNI rats. Results showed that ICI BMSCs slightly improved the erectile function more efficiently than those in the control group (P = 0.060). By comparison, the combination of PPI and ICI significantly improved erectile function. After stem cell therapy was performed, the number of nNOS-positive nerve fibres increased significantly in the PPI + ICI group. The smooth muscle/collagen ratio also increased greatly after stem cell therapy was performed in ICI and PPI + ICI groups. You suggested that ICI BMSCs could recover penile erection by decreasing corporeal smooth muscle deterioration and collagen deposition, and PPI BMSCs recovered erectile function by inducing the regeneration of nNOS-containing nerve fibres.

### Improving the efficacy of stem cell transplantation

Further efforts were devoted to investigate the transplantation using acellular scaffolds to locate more stem cells in the MPG or growth factors modification to enhance stem cell activity. Six studies from the Catholic University of Korea, along with other studies, explored the improvements of acellular scaffolds, growth factor modifications or PED-5i administration. These studies also achieved ideal therapeutic effects.

Kendirci [8] reported the effects of non-haematopoietic adult bone marrow stem/progenitor cells isolated by p75 NGF receptor (p75dMSCs) on CNI rats. Results suggested that basic fibroblast growth factor (bFGF) and NGF secreted by p75dMSCs protected the cavernous nerve after injury, and the erectile function recovery of the p75dMSC group was highly enhanced compared with that of the BMSCs group.

Lin [10] reported the effects of ADSCs seeded with an allogeneic adipose matrix to bridge the nerve gap on the rats of cavernous nerve resected. Three months after transplantation, S100 and nNOS were expressed and mainly co-localised near the MPG on the seeded matrices, indicating the extension of the cavernous nerve axon. The group of ADSCs seeded with an allogeneic adipose matrix exhibited a clear trend toward functional recovery, but this group did not show statistical significance because of large variations (P = 0.07).

Kim [13] reported the effects of BMSCs mixed with Matrixen, a kind of collagen-based biocompatible polymer, and administered to the MPG of rats with cavernous nerve crushed. Four weeks after transplantation, erectile function, PKH-26-labelled BMSCs, expressions of endothelial nitric oxide synthase (eNOS) and nNOS were significantly increased in BMSCs/Matrixen group compared with those in the BMSCs group. The author suggested that Matrixen might prevent the distribution of BMSCs after administration.

Kim [14] also reported the effects of BMSCs infected with recombinant adenoviruses expressing human BDNF on CNI rats. The intracavernosal pressure/mean arterial pressure (ICP/MAP), cavernous smooth muscle content and expressions of eNOS and nNOS were all significantly increased in the BMSC-BDNF group compared with those of BMSCs group.

Kovanecz [15] from Los Angeles Biomedical Research Institute reported the combined effects of low-dose sildenafil and mouse SkMSCs on rats with cavernous nerves resected. Results showed that SkMSCs could improve erectile function (drop rate) and increase nNOS, BDNF, smooth muscle/collagen ratio and α-SMA expression in corporal tissue sections. SkMSCs could also reduce collagen content in the penile shaft. However, the combination of SkMSCs with a very low dose of sildenafil did not enhance the recovery of erectile function.

Piao [16] reported the effects of ADSCs and BDNF-immobilised poly-lactic-co-glycolic (PLGA) membrane on rats with CNI. ICP/MAP, smooth muscle/collagen ratio, phospho-eNOS protein and nNOS expressions were significantly increased in the ADSCs-BDNF/PLGA group compared with those of ADSCs group or BDNF/PLGA group.

Jeong [19] reported the combined effects of udenafil and ADSCs-BDNF/PLGA on rats with CNI. Results showed that the udenafil-induced increase in nNOS expression was not statistically significant, but udenafil-induced increase in vascular endothelial growth factor (VEGF) expression was statistically significant. By contrast, the increase in VEGF expression in the ADSCs-BDNF/PLGA group was not statistically significant, but the increase in nNOS expression was statistically significant. ADSCs-BDNF/PLGA/udenafil treatment significantly increased nNOS expression, VEGF expression and cGMP level compared with udenafil or ADSCs-BDNF/PLGA treatment.

Kim [22] reported the effects of ADSCs and nerve growth factor (NGF)-incorporated hyaluronic acid-based hydrogel. Four weeks after transplantation, the density of co-stained ADSCs in the cavernous nerve was significantly increased in the ADSCs/NGF-hydrogel group compared with that of ADSCs group. The ICP/MAP, α-SMA and expression of eNOS were significantly increased in the ADSCs-NGF/hydrogel group compared with those of ADSCs group or NGF/hydrogel group.

Ying [25] reported the combined effects of ADSCs and autologous vein graft on rats with cavernous nerves resected. Three months after transplantation, the combined treatment improved the recovery of erectile function, yielded a larger nNOS positive area and increased smooth muscle/collagen ratio compared with the ADSCs treatment.

Lee [27] reported the combined effects of bFGF-hydrogel and ADSCs-BDNF/PLGA. Erectile function, smooth muscle/collagen ratio, nNOS content, α-SMA expression and cGMP level were significantly increased in the combined treatment group compared with those of the bFGF-hydrogel group or the ADSCs-BDNF/PLGA group.

### Special stem cells

In addition to ADSCs and BMSCs, various special stem cells, including adipose-derived stromal vascular fraction (SVF), bone marrow mononuclear cells (BMMNCs), skeletal muscle-derived stem cells (SkMSCs), NESCs, CD34/CD73-double-positive testis-derived stem cells (TDSCs) and bone marrow derived CD133^+^ cells (BMCD133^+^) were also used.

Fall [6] from Henri Mondor Teaching Hospital, France reported BMMNCs therapy on rats with CNI. BMMNCs include mesenchymal stem cells, endothelial progenitor cells and haematopoietic stem cells. Fall’s research showed that BMMNCs injection decreased the number of apoptotic cells, accelerated the normalisation of nNOS and eNOS, and partially restored erectile responses in five weeks.

Qiu [18] demonstrated that uncultured autologous SVF, including a large population of stem cells, endothelial and smooth muscle progenitor cells, injected immediately or four weeks after cavernous nerve crush injuries occurred significantly improved erectile function and increased the expressions of nNOS and neurofilament in dorsal penile nerves compared with those of the vehicle group. Smooth muscle/collagen ratio was also significantly improved in the cavernous body compared with the vehicle group.

Kim [5] from the University of Pittsburgh School of Medicine and Woo [11] from the Catholic University of Korea reported the effects of SkMSCs on rats with cavernous nerves transected. Kim found that erectile function was improved in the sham-injected group two and four weeks after SkMSCs were injected into the penis. The percent area of PGP9.5 staining was significantly greater in SkMSCs-injected penis than in sham-injected penis in two and four weeks. The increase in PGP9.5 neuronal staining suggested that SkMSCs protected the penile nerve from atrophy after cavernous nerve transection was performed. Woo found an acceptable survival of PKH-26-labelled SkMSCs and high expression of cGMP in the cavernous body. He also found that erectile function was improved four weeks after transplantation. The increased cGMP expression suggested that SkMSCs protected the penile nerves or muscle from atrophy after cavernous nerve transection was performed.

Choi [23] from CHA University, Seoul, Korea successfully isolated the human TDSCs. The study showed erectile functions of rats with CNI recovered with the treatment of TDSCs, which was considered as one of the characteristics of TDSCs.

Miyamoto [26] from Hiroshima University, Japan reported that the transplanted BMCD133+ accelerated erectile functional recovery. The nNOS positive area in the BMCD133+ therapy group was larger than that in the control group.

These studies evaluated the fates, effects and action mechanism of stem cells transplanted, including special stem cells. At the same time, they tried to improve the therapeutic effects. To evaluate the true value of such improvements, we defined the influential factors and evaluated the criteria that could be used for further analysis.

### Influential factors and evaluation criteria

Studies evolved in our analysis presented numerous differences. These differences among studies should also be extensively investigated to elucidate improvement strategies in the future. The major differences among those studies were considered as influential factors.

Many elements, including CNI models, follow-up time, stem cell species, stem cell sources, stem cell numbers, markers and delivery approaches, growth factor modifications, stimulations used when measuring intracavernosal pressure (ICP) and so on, may have influences on the outcomes of stem cell transplantation on rats with CNI.

Among the 24 studies, cavernous nerves were kept continuous in sixteen rat models: fourteen showed cavernous nerve crush injuries; one displayed cavernous nerve stretch injury; and one described cavernous nerve radiation. Cavernous nerves were transected in seven rat models. The mechanism by which cavernous nerve injury was caused was unclear in one study. After the cavernous nerve was damaged (ranging from crushed neuropraxia to axonal breakage), the nerve was unable to release NO to the cavernous body to induce engorgement, and the penile tissue became chronically hypoxic, leading to smooth muscle cell apoptosis and subsequent tissue fibrosis [28, 29]. A significantly spontaneous recovery of erectile function was observed six months later after cavernous nerve crush injuries occurred [30], and this type of recovery should be quite weak in axonal breakage models. Different CNI models should exhibit different recovery mechanisms, but erectile functions were not significantly different between crushed cavernous nerves and neurotomy 10 days after the onset of initial injury, as a research revealed [31]. Whether stem cell transplantation with similar pathological lesions have displayed different therapeutic mechanisms and effects on different CNI models is still unclear.

Follow-up periods varied from two weeks to seventeen weeks but most commonly from one month (in 14 studies) to three months (in six studies). In one study, the therapeutic effects of stem cells were detected as early as two weeks after stem cells were transplanted to rats with cavernous nerves transacted [5]. In another study, no evident erectile function recovery was observed three weeks after stem cells were transplanted to rats with ablated cavernous nerves until five weeks [6]. Moreover, almost no difference was found between immediate and delayed (four weeks later) stem cell injection groups on rats with cavernous nerve crush injuries [18]. Other studies repeatedly showed that ICI stem cells disappeared from the penis in a rapid and time-depended manner. Since stem cells disappear rapidly, whether a prolonged follow-up time is advisable should also be clarified.

Among the 24 published articles, five involved autotransplantation, eleven focused on allotransplantation and nine discussed heterotransplantation; only one study compared the migration of autologous and allogeneic stem cells [9]. Almost no study described immunological rejection in different sources of transplanted stem cells in rats with CNI. Stem cells lack the expression of major histocompatibility complex II, therefore, stem cells elicit no immune reaction when transplanted allogeneically or xenogeneically [32]. Considering large numbers of stem cells disappear after transplantation, researchers should evaluate the effects of rejection induced by different sources of stem cell transplantation on rats with CNI.

Cultured stem cells, including ADSCs in twelve studies and BMSCs in four studies, were used. Some studies showed that ADSCs exhibited a higher proliferation rate [33] and a lower percentage of apoptotic and senescent cells than BMSCs [34]. Other researchers demonstrated that BDNF released from the induced BMSCs were almost two fold greater than those from the induced ADSCs [35]. In addition to being easily accepted, less trauma and abundant supply, ADSCs are more suitable for clinical applications. With the increasing applications of ADSCs, the effects of ADSCs and BMSCs should be compared.

In previous studies, SVF and BMMNCs were used as uncultured stem cells. These uncultured stem cells prevented contamination and cytometaplasia while or after cells were cultured, resulting in great changes in future stem cell transplantation. The therapeutic effects of uncultured stem cells should be compared with those of cultured stem cells to elucidate the application potential of SVF and BMMNCs.

Among the 24 published articles, a questionable number of stem cells (0.5 × 10^3^) were used in one study [4], an unclear number of stem cells were presented in another study [10] and uncultured stem cells were used in two studies [6, 18]. In 17 of these published articles, 1 × 10^6^ stem cells were used. In the other studies, the number of transplanted stem cells was 0.5 × 10^6^, 1 × 10^7^ and 2 × 10^7^ respectively. The subgroup analysis of the numbers of stem cells was unlikely feasible in the current research.

Labelling and tracking stem cells are very important because limited stem cells can be detected after transplantation as revealed in many studies. Stem cell markers include lipid-soluble markers, such as CM-DiI (used in one study) and PKH-26 (in six studies), and nuclear labelling markers, such as EDU (in five studies), Laz (in one study), GFP (in two studies) and CELL STALKER (in two studies). The stem cell markers used in seven other studies were unclear or none. Lipid-soluble markers are believed to provide false-positive identification and affect stem cell survival [36]. Detectable stem cells after transplantation are very few, thus the therapeutic effects of stem cell markers should be considered.

Studies have shown that different delivery approaches may require different mechanisms but elicit similar effects on erectile function recovery [4, 20, 21]. For example, ICI stem cells decrease the deterioration of corporeal smooth muscles and the deposition of collagen. PPI stem cells induce the regeneration of nNOS-containing nerve fibres. Furthermore, the stem cells of ICI can migrate to the MPG [9, 12]. The delivery approaches of PPI, MPG administration or injured cavernous nerve covering (ICNC) share common characteristics, such as adjacent location, poor blood supply and similar purpose in nerve connection restoration (For convenience of description, PPI, MPG administration or injured nerve covering are all expressed as PPI in the following). However, no evidence has been provided to show that the stem cells of the surrounding periprostatic cavernous nerves/MPG migrate to the cavernous body. The similar recovery of erectile function between two delivery approaches may be a coincidence. The exact pathological changes in the cavernous body need further evaluation.

In other studies, acellular scaffolds, including allogeneic adipose matrix, Matrixen, vein graft, PLGA membrane, hyaluronic acid-based hydrogel and alginate gel sponge sheet, have been used to locate the stem cells in the MPG or in an injured cavernous nerve to enhance the nerve regeneration. However, these methods limit the migration of stem cells to the cavernous body at the same time. Whether cells recovery in the cavernous body is similar remains unclear.

Erectile functions are evaluated in terms of peak ICP or ICP/MAP. By peak ICP, the interference of systemic blood pressure variations cannot be eliminated, thus ICP/MAP is more reliable than peak ICP. Penile drop rate is also less accurate than ICP/MAP. In 22 studies ICP or ICP/MAP was evaluated via electrical stimulation. In two studies, erectile functions were evaluated via papaverine administration. While, personal differences in pharmacokinetics are factors that need to be considered. Thus, ICP/MAP is the optimal evaluated criterion of the erectile function in stem cell transplantation for rats with CNI.

Besides the evaluation of erectile function, some histological assessments were also performed to evaluate the structural changes related with the functional improvement. In five studies, the numbers of vascular endothelial cells were evaluated by eNOS; in one study, the number of vascular endothelial cells was evaluated by vWF; in seven studies, the numbers of smooth muscle cells were evaluated in terms of α-SMA. The numbers of nerve fibres were evaluated in terms of nNOS, neurofilament and NADPH-d in 15, two and two studies, respectively. In one study, fibrosis was evaluated in terms of collagen III. In ten studies, smooth muscle/collagen ratios were evaluated. Apoptosis was evaluated on the basis of apoptotic index and cGMP in two and four studies, respectively. cGMP is not an optimal criterion of apoptosis, but it represents that the cell function is normal to some extent. Quantitative measurements used in these studies include quantitative PCR, Western-blot analysis, immunohistochemistry and so on.

### Meaning of meta-analysis

The action mechanisms of stem cell transplantation focus on the paracrine action of stem cells, because few stem cells can be detected after transplantation, and almost no direct evidence supports the theory that transplanted stem cells have differentiated into vascular endothelial cells, smooth muscle cells or nerves [28, 29]. At the same time, some studies confirmed the expressions of growth factors after stem cells were transplanted [8, 14, 15, 19, 26]. In other studies, growth factor expressions modifications could improve the therapeutic effects of stem cells [4, 8, 14, 16, 19, 22, 27]. These studies showed that growth factors were implicated in stem cell transplantation on rats with CNI.

Paracrine mediators of stem cells are either expressed or released depending on the microenvironment after injury [37]. In rats with CNI, microenvironments differ between a cavernous body and MPG. Apoptosis and fibrosis occur in the cavernous body, while Wallerian degeneration occurs in MPG [28, 29]. Thus, the action mechanisms of stem cells vary in different microenvironments. Stem cells located in different target tissues possibly undergo different mechanisms. However, studies have shown that the improvements of ICP/MAP or ICP are almost the same when stem cells are injected in either the cavernous body or around the impaired cavernous nerves. Thus the erectile function should be evaluated not only by ICP/MAP or ICP, bur also by histological assessments.

If nerve injury is the primary cause, stem cells should be placed in the MPG or injured nerves to restore the innervation of cavernous body. This procedure is possibly reasonable and reliable. Although CNI upregulates SDF-1 expression in the MPG and induces ICI stem cells to migrate to MPG, the number of stem cells recruited in the MPG remains very few [9, 12]. Stem cell transplantation via PPI is believed to be able to improve nerve regeneration, for locating numerous stem cells in the MPG or injured nerves. However, whether the improvements result in a decrease in the number of cells recovering in the cavernous body is rarely investigated. Hence, a meta-analysis of different delivery approaches on the therapeutic effects of stem cells should be considered.

Stem cells transplanted with modified growth factors [4, 8, 14, 16, 19, 22, 27] or combined with PDE-5i [15, 19] represent another example of improvements to promote therapeutic effects. High-level growth factors, such as BDNF and NGF, promote nerve regeneration. The rehabilitation of erectile function depends on nerve regeneration and cell apoptosis inhibition in the cavernous body. So before innervation is completely finished, vascular cells in the cavernous body, such as smooth muscle cells and endothelial cells should be protected.

Studies have shown that growth factor modification of stem cells can significantly improve the recovery of erectile function. However, comparisons are seldom made among the effects of different growth factor modifications on the vascular cell recovery in the cavernous body. Therefore, a meta-analysis of the improvements in the therapeutic effects on growth factor modifications should be conducted.

In our research, most of the studies involved are valid and relevant for future pre-clinical applications. Some of these studies may suggest improvements for future stem cell transplantation on rats with CNI. Hence, influential factors and improvements affecting stem cell transplantation in ED rats should be evaluated. We believe that the following meta-analysis of these studies may be helpful to design future pre-clinical and clinical studies.

## Meta-Analysis

### Literature search

We searched Pubmed and EBSCO for relevant studies published before April 30, 2014 by using the following search terms: “rat AND (erect* OR impoten*) AND (nerve OR neuro*) AND (injury OR crush OR regeneration OR clamp OR dissect*) AND (stem OR progenitor OR “bone marrow” OR “cell transplantation”)”. Non-English language papers, news, reviews, discussions, comments, abstracts, conference papers and unpublished studies were not considered. The received studies were carefully examined to exclude potentially overlapping data. The complete search strategy is available upon request.

### Selection criteria

Eligible studies should satisfy the following criteria: 1) randomized controlled trial, comparative study or controlled trial; 2) ED rat model with CNI; 3) use of stem cells, stromal cells, mesenchymal cells, progenitor cells, SVF or BMMNCs; and 4) with a minimum follow- up period of 1 week.

The exclusion criteria are listed as follows: 1) abnormal distribution of ICP/MAP or ICP databases; 2) no evaluation of erectile function; 3) cavernous nerve injured by radiation; 4) papaverine administration during erectile function evaluation; 5) unclear CNI methods; and 6) conflicting databases, such as those with a condition in which MAP did not differ significantly among groups, ICP and ICP/MAP changed with a different pattern, an observation indicator had two different databases or standard deviations increased with a proportional decrease of the mean.

### Quality Assessment

The methodological quality of the studies was assessed independently by two authors, Hai-tao Shan and Peng-zhi Chen. In case of missing details, the corresponding author was contacted. The assessment of research quality should include the following three fields.

An acceptable study design (3 points) should exhibit the following characteristics: 1) adequate sample size of ICP/MAP or ICP (single side α = 0.05, 1-β = 80%; not, 0 point; yes, 1 point); 2) randomisation (unclear or no mention, 0 point; yes, 1 point); and 3) blind assessment of outcome (unclear or no mention, 0 point; yes, 1 point).

Interference factors (4 points) were as follows: 1) stem cell label marker(unclear or no mention, 0 point; lipid-soluble markers, such as CM-DiI and PKH-26, 0.5 point; nuclear labeling such as EDU, lacZ and GFP, 1 point); 2) stem cell phenotype identity (unclear or no mention, 0 point; yes, 1 point); 3) erectile function measurement (ICP, 0.5 point; ICP/MAP 1 point); 4) structural changes in the penile environment report (none, 0 point; one method among the following: PCR, Western-blot analysis and immunohistochemistry, 0.5 point; two or more, 1 point).

Peer evaluation (1 point) was based on impact factor (SCI was not induced, 0.5 score; 0> and ≤3, 0.5 score; and >3, 1 point).

The studies of quality analysis with a total score of >4 were included in our meta-analysis.

### Data extraction

Two authors, namely, Hai-tao Shan and Tao Zhang, extracted data using a standardised data extraction sheet. This procedure was conducted independently and in duplicate. Disagreements were resolved by consensus. Data were obtained by measuring the chart on the condition of no responding from the author. The following information was extracted from the articles: 1) characteristics of the studies and 2) cell marker expressions of nNOS, eNOS, α-SMA and ICP or ICP/MAP.

### Data analysis

Stata SE 12 was used to analyze outcome data. Our primary outcome was the standardized mean difference in erectile functions between the stem cell treatment groups and control groups. On the basis of a clinical point of view, we performed the subgroup analyses as mentioned above. In case of multiple treatment groups next to one control group within one trial, the number of rats in the control group was divided equally by the number of treatment groups.

Continuous variables were reported as standard mean differences with 95% confidence intervals (CIs) between cell-treated groups and control groups. A fixed-effect model was used in the analysis without heterogeneity, otherwise, a random-effect model was utilised. The level of heterogeneity across studies was estimated by chi-square tests; *I* ^2^ > 50% was considered to indicate significant heterogeneity between subgroups. P < 0.05 was considered statistically significant; two-sided significance values were reported throughout our meta-analysis.

## Results

### Studies included

**Fig 1:**
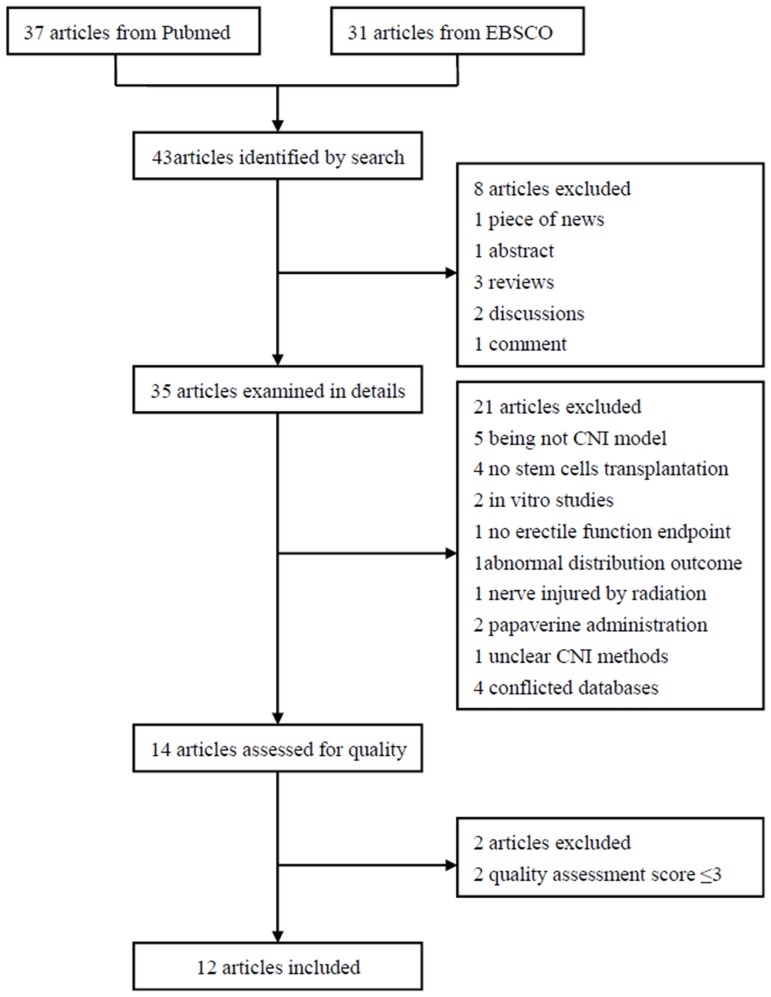
Flowchart of enrolled studies on stem cell therapy in ED rats with CNI.

After conducting electronic database search and manual search, we obtained 37 articles from PubMed and 31 articles from EBSCO. A total of 43 potentially relevant publications were obtained. After identifying relevant studies, we found that 12 articles were eligible for meta-analysis and a total of 632 rats were included. Among these rats, 319 were enrolled in our meta-analysis (Fig 1).

### Methodological quality

The quality scores of 12 studies are >4. The impact factors of 11 articles are >3. Strictly speaking,the sample sizes of only 6 studies are adequate. Among the studies, 12 reported the randomisation of rats and/or blinding outcome assessment. The stem cell-labelling markers in 5 studies were soluble in lipid; in another 5 studies, nuclear-labelling markers were used. In 5 studies, stem cells were subjected to phenotype identification. In all of the 12 studies, erectile functions were evaluated in terms of ICP/MAP. 11 of the 12 studies reported the structural changes in the penile environment. Among these studies, 7 reported the outcomes measured by at least two methods (Table 3).

**Table 3:** Methodological quality assessment.

Studies		Peer evaluation (1score)	Study design (3 scores)		Confining the interference factors (4 scores)				Outcomes
Year	First Author	Impact Factors	Sample Size	Blind & Random	Cells Label	Phenotype Identify	Erectile Function	Structural Changes	Scores
2004	Bochinski [4]	1	0.5	0	1	0	0.5	0.5	3.5
2006	Y Kim [5]	0.5	0	1	1	0	0.5	0.5	3.5
2009	Fall [6]	1	0	1	0.5	0	1	1	4.5
2010	Albersen [7]	1	0	1	1	0	1	0.5	4.5
2010	Kendirci [8]	1	1	1	1	1	1	1	6
2012	Piao [16]	1	0.5	2	0.5	0	1	1	5.5
2012	Qiu [18]	1	0.5	2	0	0	1	0.5	5
2013	Jeong [19]	0.5	1	2	0	0	1	0.5	5
2013	You [20]	1	1	2	1	1	1	1	7
2013	IG Kim [22]	1	1	1	0.5	0	1	0.5	5
2013	Choi [23]	1	0	1	0.5	1	1	0.5	5
2013	Ying [24]	0.5	1	1	0	1	1	1	5.5
2014	Miyamoto [26]	1	1	0	0	1	1	1	5
2014	Lee [27]	1	0.5	1	0.5	0	1	1	5

### Characteristics of the studies enrolled in the meta-analysis

The cavernous nerves were discontinuous in two studies but continuous in ten studies. The follow-up time in three studies was three months; in other studies, the follow-up time was approximately one month. Two, three and seven studies focused on autotransplantation, allotransplantation and heterotransplantation respectively. In four studies, stem cells were derived from the bone marrow; in eight studies, stem cells were derived from adipose tissues. Uncultured stem cells were used in two studies; in other studies, cultured stem cells were used. In six studies, stem cells were injected into the cavernous body; in seven studies, stem cells were injected into periprostatic cavernous nerves/MPG surrounding. In six studies, acellular scaffolds were used. In five other studies, growth factor modification or PDE-5i administration was performed.

In CNI models, adult SD rats were used. Many criteria including eNOS, nNOS, α-SMA, collagen III, cGMP and so on, were measured to evaluate the histological changes of the cavernous body tissue. nNOS was the most common nerve fibre marker and represented the regeneration of cavernous nerves. α-SMA and eNOS were the most common smooth muscle cell and vascular endothelial cell markers representing the recovery of vascular cells. In six, three and eight studies, the expressions of α-SMA, eNOS and nNOS were evaluated respectively (Table 4).

**Table 4:** Characteristics of Studies Enrolled.

Studies		Rats			Group		Immunohistochemistry
Year	Author	Spices	Age	Weight(g)	Group	Sample size/group	Quantitatively evaluated outcomes
2009	Fall [6]	Fisher	Adult	170–200	Normal vs CNI vs CNI+BMMNCs ICI (3week vs 5week)	40/4	eNOS, nNOS, apoptotic index
2010	Albersen [7]	SD	12w	No mentioned	Normal vs CNI vs CNI+ADSCs ICI vs CNI+lystate	32/4	a-SMA, collagen III, nNOS
2010	Kendirci [8]	SD	Adult	300–350	Normal vs CNI+PBS vs CNI+ fibroblasts vs CNI+BMSCs ICI vs CNI+P75MSCs ICI	40/5	VEGF, NGF, BDNF, FGF, IGF
2012	Piao [16]	SD	Adult	250–300	Normal vs CNI vs CNI+ADSCs ICNC vs CNI+BDNF+PLGA vs CNI+ADSCs ICNC +BNDF+PLGA	50/5	nNOS, eNOS, cGMP smooth muscle/collagen,
2012	Qiu [18]	SD	12w	No mentioned	Normal vs CNI vs CNI+ vehicle vs CNI+SVFICI (immediate vs delayed)	89/4	a-SMA, neurofilament, smooth muscle/collagen, nNOS
2013	Jeong [19]	SD	Adult	250–300	Normal vs CNI vs CNI+ udenafil vs CNI+ADSCs ICNC+BDNF+PLGA vs CNI+ADSCs ICNC+BDNF+PLGA+ udenafil	30/5	VEGF, nNOS, cGMP, smooth muscle/collagen, a-SMA,
2013	You[20]	SD	8w	250	Normal vs CNI vs CNI+ADSCs ICI vs CNI+ fibrin sealant vs CNI+ADSCs PPI+ fibrin sealant vs CNI+ADSCs ICI+ ADSCs PPI+ fibrin sealant	60/6	a-SMA, nNOS
2013	IG Kim [22]	SD	8-10w	No mentioned	Normal vs CNI vs CNI+ADSCs ICNC vs CNI+ NGF-hydrogel vs CNI+ADSCs ICNC+ NGF-hydrogel	25/5	eNOS, a-SMA
2013	Choi [23]	SD	12w	No mentioned	Normal vs CNI vs CNI+BMSCs PPI vs CNI+TDSCs PPI	32/4	none
2013	Ying [24]	SD	4m	Normal	Normal vs CNI vs CNI+ADSCs ICI	130/3	myelinated axons, nNOS, smooth muscle/collagen, NADPH-d
2014	Miyamoto [26]	athymic	8w	230–250	Normal vs CNI vs CNI+ alginate gel sponge Sheet vs CNI +BMCD133 ICNC + alginate gel sponge Sheet	28/4	VEGF, NGF, blood vessel density, nNOS
2014	Lee [27]	SD	8-10w	No mentioned	Normal vs CNI vs CNI+ADSCs+ BDNF+PLGA vs CNI+bFGF-hydrogel ICI vs CNI+ADSCs ICNC+ BDNF+PLGA +bFGF-hydrogel ICI	75/5	smooth muscle/collagen, a-SMA, cGMP

SkMSCs: skeletal muscle-derived stem cells; BMMNCs: bone marrow mononucleated cells; ADSCs: adipose tissue-derived stem cells; BMSCs: bone marrow stem Cells; SVF: adipose-derived stromal vascular fraction; TDSCs: testis-derived stem cells; BMCD133+: Bone Marrow Derived CD133+ Cells. ICI: intracavernousal Injection; ICNC: Injured cavernous nerve cover; PPI: periprostatic implantation.

### Meta-analyses

**Fig 2:**
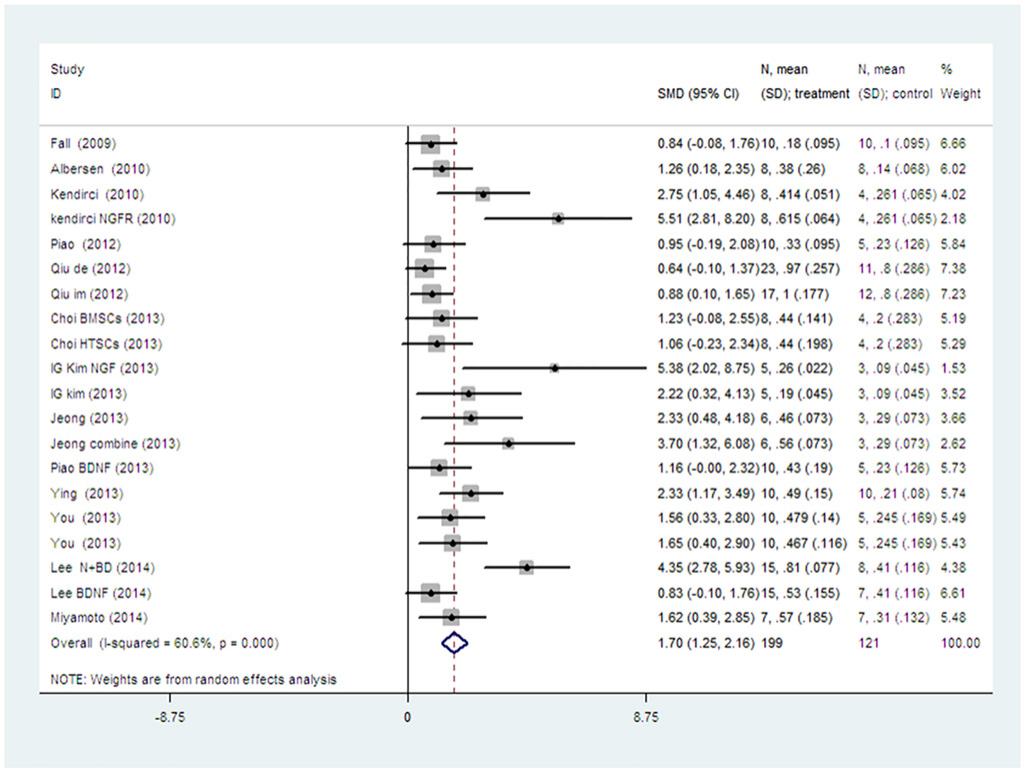
The random-effect model forest plot graph shows the standard mean differences (SMD), 95% confidence intervals (CI) while the meta-analysis of ICP/MAP was evaluated and the number of cases (n), mean and standard deviation (SD) in the stem cell treatment and control groups.

The pooled analysis of all the studies showed a significant difference of erectile functions between stem cell transplantation group and control group (SMD 1.701, 95% CI = 1.245 to 2.158, P < 0.001, *I* ^2^ = 60.6%) with heterogeneity (chi-square = 48.25, p<0.001; Fig 2).

**Fig 3:**
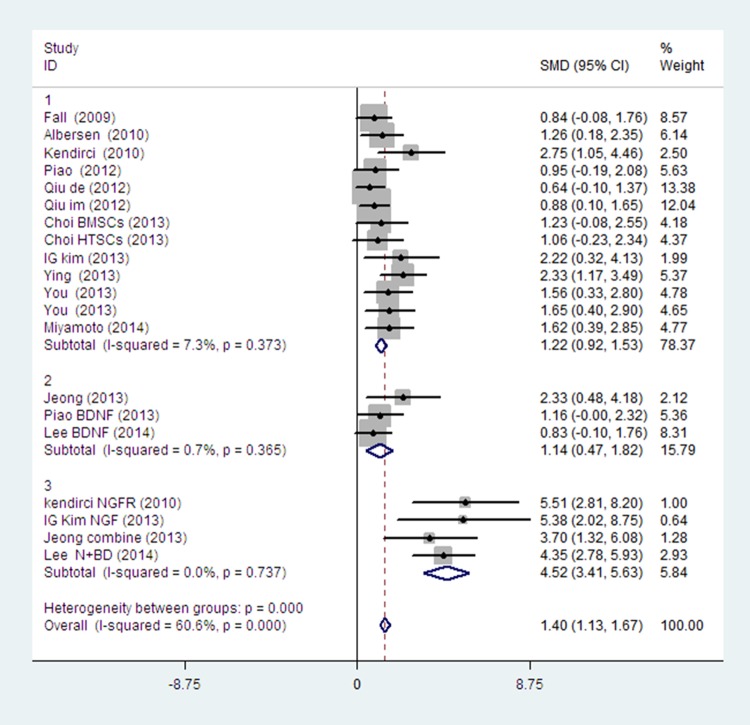
The fixed-effect model forest plot graph shows there is no significant difference between the stem cells with BDNF modification (subgroup 2) or without growth factor modification (subgroup1). But subgroups with NGF modification, p75LNGFR isolation, BDNF+ udenafil or BDNF+bFGF (subgroup3) can get better effects than those modified by BDNF only (subgroup2) or without any modification (subgroup1). (subgroup 1 vs. subgroup 3 p<0.001; subgroup2 vs. subgroup 3 p<0.001).

Firstly, we took the growth factor modification as a source of heterogeneity. A subgroup analysis showed that BDNF modification had limited influence on improving the recovery of erectile function; by contrast, NGF modification, p75LNGFR isolation, combination of BDNF and udenafil or bFGF showed a significant improvement of erectile function. (Fig 3).

**Fig 4:**
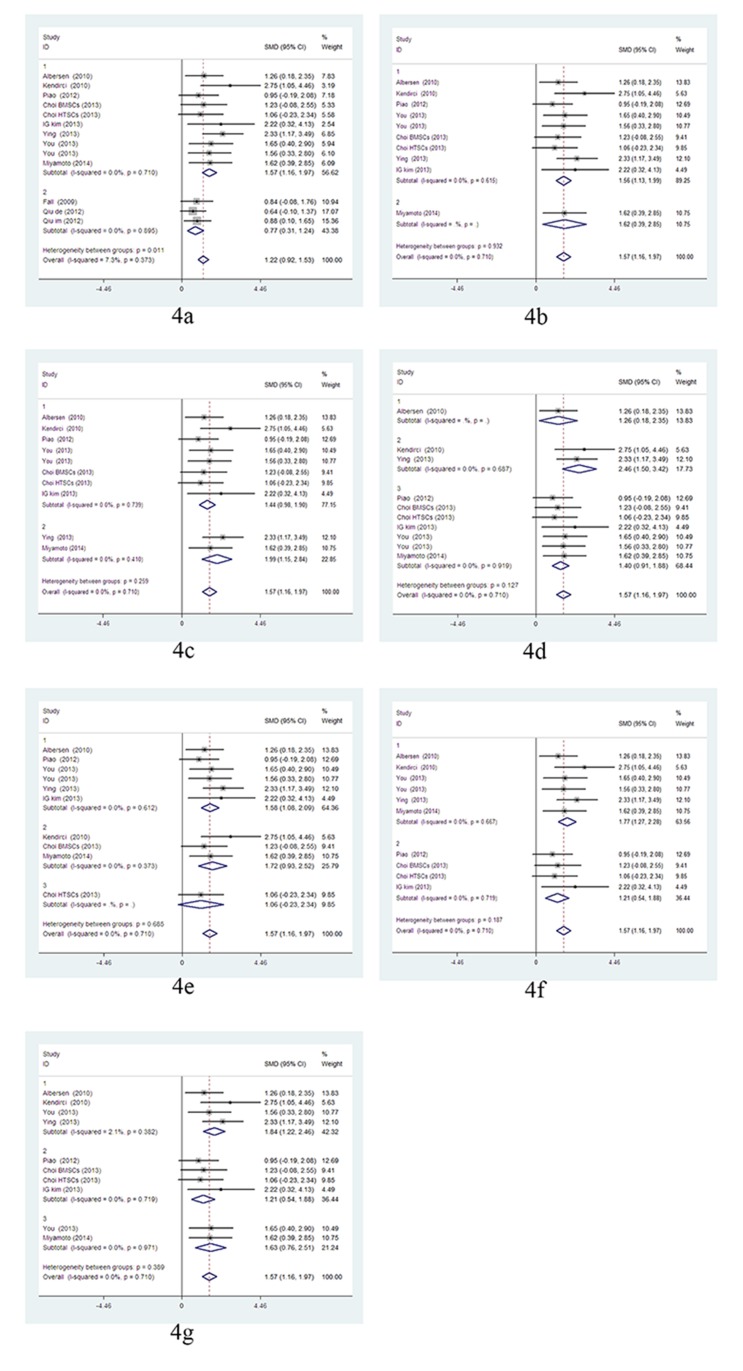
The fixed-effect model forest plot graphs show the uncultured stem cells (BMMNCs and SVF) are significantly less effective than cultured stem cells(4a), while there is no significant difference among the following subgroups: CNI models(4b), following time(4c), stem cells species(4d), cell sources(4e), labels(4f) and delivered approaches(4g). Fig 4a: cultured stem cells (subgroup1) vs. uncultured stem cells; (subgroup 2), p = 0.011. Fig 4b: cavernous nerve continuous (subgroup1) vs. cavernous nerve discontinuous (subgroup2). Fig 4C: Follow-up time: one month (subgroup1) vs. three months (subgroup2). Fig 4d: autotransplantation (subgroup1) vs. allotransplantation (subgroup2) vs. heterotransplantation (subgroup3). Fig 4e: ADSCs (subgroup1) vs. BMSCs (subgroup2) vs. TDSCs (subgroup3). Fig 4f: nuclear labelling (subgroup1) vs. lipid-soluble markers (subgroup2). Fig 4g: ICI (subgroup1) vs. PPI (subgroup2) vs. PPI + acellular scaffolds (subgroup3).

Secondly, within the subgroups of stem cell transplantation without any growth factor modifications, the subgroup analysis showed uncultured stem cells (BMMNCs and SVF) were significantly less effective than cultured stem cells (p = 0.011), and there were no significant differences of erectile functions among the following subgroups: cavernous nerve discontinuous vs. continuous; follow-up time of one month vs. three months; autotransplantation vs. allotransplantation vs. heterotransplantation; stem cell sources of bone marrow vs. adipose tissue; lipid-soluble markers vs. nuclear markers; and delivery approaches, particularly ICI vs. PPI vs. PPI+acellular scaffolds. But there were heterogeneities in the subgroups of 3 months follow-up (*I* ^2^ = 56.0%) and allotransplantation (*I* ^2^ = 66.1%). After dropping the studies of uncultured stem cells, the outcomes of subgroup analysis showed similar results without heterogeneity. (Fig 4).

**Fig 5:**
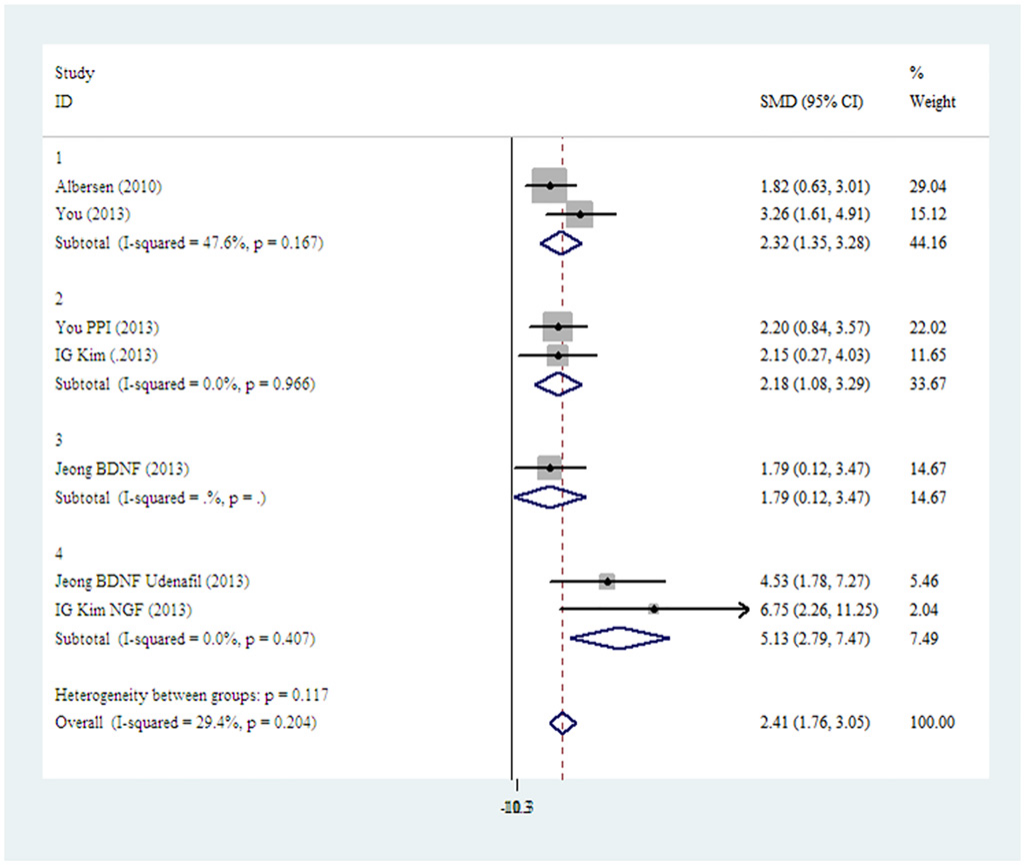
The fixed-effect model forest plot graph shows there is no significant difference between different delivery approach of ICI (subgroup1), PPI+ acellular scaffolds (subgroup2) or PPI+ acellular scaffolds+BDNF (subgroup3) in the a-SMA expression; the NGF, BDNF+ udenafil, BDNF+ bFGF subgroup(subgroup4) can restore the a-SMA expression more effectively than the other subgroups (subgroup 1 vs. subgroup 4 p = 0.029; subgroup 2 vs. subgroup 4 p = 0.026; subgroup 3 vs. subgroup 4 p = 0.023).

To further investigate the mechanisms of stem cell transplantation, we performed a pooled analysis on immunohistochemistry of α-SMA expression four weeks after cultured stem cells transplantation. We found that there was no significant difference between different delivery approaches of ICI and PPI+ acellular scaffolds. BDNF administration showed no advantage in improving α-SMA expression, but NGF, BDNF combined with bFGF or udenafil could significantly restore α-SMA expression more than BDNF alone (Fig 5).

**Fig 6:**
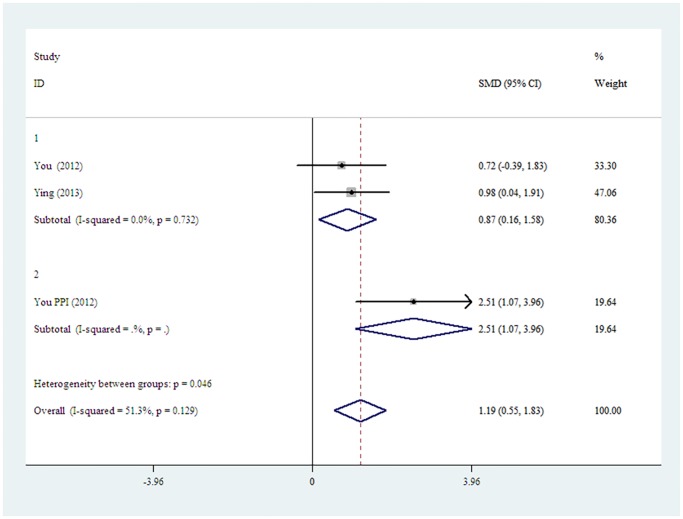
The fixed-effect model forest plot graph shows PPI+ acellular scaffolds subgroup (subgroup2) is significantly higher than the ICI subgroup (subgroup1) in the restoring of nNOS expression, (p = 0.046).

A meta-analysis on Westen-blot of nNOS expression four weeks after cultured stem cells transplantation was performed. The results showed that stem cells delivery approach of PPI+ acellular scaffold exhibited a significantly higher expression of nNOS than ICI (Fig 6).

## Discussion

Our analysis is composed of data from 12 published pre-clinical studies involving rats treated with stem cells to investigate the effects of stem cell therapy for ED rats with CNI. The main findings are described as follows: 1) stem cell therapy can improve erectile function because of a significant increase in ICP/MAP; 2) uncultured stem cells (BMMNCs and SVF) are less effective than cultured stem cells; 3) without growth factor modifications the following elements have no effect on therapeutic activity of stem cells: cavernous nerves being continuous or discontinuous; follow-up time of one month or three months; autotransplantation, allotransplantation or heterotransplantation; stem cell source being bone marrow or adipose tissue; lipid-soluble labels or nuclear labels; delivery approach of ICI or PPI with or without acellular scaffolds; 4) PPI+acellular scaffolds are advantageous for nNOS expression, but not for α-SMA expression; 5) NGF modification, p75LNGFR modification and BDNF combined with udenafil or bFGF are more effective than BDNF alone on erectile function recovery.

We found no significant difference among the therapeutic effects of stem cells in different CNI models. The similar pathologic changes in both kinds of CNI models possibly resulted in a similar paracrine activity of stem cells and similar recovery of erectile function, as our analysis revealed. Two meta-analysis studies on spinal cord injury have also revealed that the therapeutic effects of stem cells do not significantly differ between the methods of injury induction [38, 39]. Even so, the effects of stem cell transplantation on different CNI models require further studies.

The therapeutic effects between the follow-up time of one month and three months did not significantly differ. With prolonged follow-up time, abundant stem cells disappeared rapidly. One month after transplantation, only a few labelled stem cells could be detected. Therefore, the level of paracrine mediators from stem cells should be extremely low. The major effects of stem cell transplantation should have been completed at the beginning of the therapy. In one study, the single injection of ADSC lysate could elicit effects similar to ADSCs [7]. In another meta-analysis of spinal cord injury, therapeutic effects of stem cells were almost the same in three weeks and more than five weeks after transplantation, although the degree of injury and the recovery speed after transplantation in different models were different [40]. This result is consistent with our meta-analysis.

Our results showed that the uncultured stem cells were significantly less effective than the cultured stem cells. The number of stem cells in SVF and BMMNCs were lower than that of cultured stem cells, such as BMSCs and ADSCs [41], thus the level of paracrine mediators from uncultured stem cells should be lower. Although uncultured stem cells (SVF and BMMNCs) are relatively less effective, they exhibit a high potential for clinical applications.

Our results showed that different species, sources, labels of stem cells elicited similar effects on rats with CNI. As such, different kinds of stem cells at the initial transplantation stage should exhibit similar paracrine actions, and the rejection and cytotoxic effects of labels almost have no effects on the recovery of erectile function.

It is a coincidence that different delivery approaches elicited similar effects. Although penis is conventionally considered as a part of the urinary system, it is a vascular organ [42]. Stem cells injected into the cavernous body naturally migrate to the MPG because of the upregulation of SDF-1 expression in the MPG, however, stem cells injected around the cavernous nerves or the MPG experience difficulties in migrating to the cavernous body against a concentration gradient of SDF-1. Within our research, we found PPI+acellular scaffolds were advantageous for nNOS expression, but not for α-SMA expression. The stem cell delivery approaches of ICI and PPI involve different active mechanisms [20, 21], thus, therapeutic effects may vary.

Various types of acellular scaffolds have been used in stem cell therapy. Scaffolds can place numerous stem cells to a target tissue and possibly get better therapeutic effect in transplantation. Researchers reported improved effects when scaffolds were applied [13, 16, 22]. Our meta-analysis showed that acellular scaffolds could promote cavernous nerve regeneration but were less effective for smooth muscle cell recovery. That explains why the subgroups with scaffolds cannot get remarkably better recovery of erectile functions than the subgroups without scaffolds.

NGF and BDNF are the common growth factors used in the enhancement of stem cell therapy for ED rats with CNI, and both of them can enhance nerve regeneration [43–45]. But in our meta-analysis BDNF alone elicited almost no effect on restoring α-SMA expression in the cavernous body. It is believed that BDNF and VEGF function via the Janus kinase/signal transducer and activator of transcription pathway and promote neurite growth in the MPG [46]. Thus, the nerve regeneration is promoted by a synergistic effect of several growth factors, not by BDNF alone.

The BMSCs isolated by p75 nerve growth factor receptor secretes significantly higher amounts of bFGF and NGF than BMSCs [8]. bFGF mediates recovery effects on injured sensory neurons and supports neurite outgrowth of transected nerves [47]. The recombinant bFGF can improve vasoreactivity in corporal tissue [48]. PED-5i has been considered as a vascular regenerative therapy for the treatment of erectile dysfunction [49]. The cavernous body and cavernous nerves were used as target tissues in the studies involving stem cell transplantation with NGF modification, p75LNGFR isolation, combination of BDNF and udenafil or combination of BDNF and bFGF. The above-mentioned subgroups can promote the recovery of smooth muscle cells and cavernous nerves, therefore, optimal effects are achieved in all of the current studies.

In brief, a small number of stem cells existing in the cavernous body after transplantation, the MPG or impaired cavernous nerves result in limited paracrine activity of stem cells and therapeutic effects. Stem cells placed in the MPG or around the impaired cavernous nerves with scaffolds can efficiently promote nerve regeneration, but limit vascular cell recovery in the cavernous body at the same time. Nevertheless, nerve and vascular regeneration can be achieved and optimal effects can be obtained by enhancing NGF, administrating BDNF with bFGF or PDE-5i simultaneously when stem cells are transplanted.

The following factors should be considered in future pre-clinical trials to obtain preferable effects. The function of nerve regeneration and vascular cell recovery should be deliberated simultaneously. The synergistic actions of multiple growth factors or agents during stem cell transplantation should be further explored before such factors are clinically applied.

### Limitations

Conclusions from this meta-analysis should be considered cautiously and should be substantiated by larger studies because of the small number of studies included in this meta-analysis and small sample sizes of animals. Moreover, these limitations restrict further analyses of vascular endothelial cells, fibrosis and apoptosis, as well as the nNOS subgroup analysis of growth factor modifications and scaffold application.

Some studies have presented outcomes by using a chart. For authors with poor responses, databases were obtained by measuring a column graph. However, the accuracy of such databases was insufficient.

### Conclusions

To the best of our knowledge, this study is the first meta-analysis to evaluate the effect of stem cell therapy on ED rat models with CNI. This analysis shows that the studies with rats are valid and can predict outcomes of pre-clinical studies. Moreover, the results show that stem cell therapy is safe and can lead to improved ICP/MAP. Future studies should simultaneously focus on nerve regeneration and vascular cell recovery. The synergistic effect of multiple growth factors or agent administration in stem cell transplantation should be considered as beneficial strategies to obtain preferable effects.
